# Differential Expression of Myogenic Regulatory Factor Genes in the Skeletal Muscles of Tambaqui *Colossoma macropomum* (Cuvier 1818) from Amazonian Black and Clear Water

**DOI:** 10.1155/2013/465727

**Published:** 2013-11-19

**Authors:** F. A. Alves-Costa, C. M. Barbosa, R. C. M. Aguiar, E. A. Mareco, M. Dal-Pai-Silva

**Affiliations:** ^1^Universidade Paulista (UNIP), Instituto de Ciências da Saúde, R. Luiz Levorato 20108, 17048-290 Bauru, SP, Brazil; ^2^Universidade Estadual Paulista (UNESP), Instituto de Biociências, Departamento de Morfologia, Laboratório de Biologia do Músculo Estriado, Distrito de Rubião Jr., s/n, 18618-000 Botucatu, SP, Brazil

## Abstract

Hypothesizing that the Amazonian water system differences would affect the expression of muscle growth-related genes in juvenile tambaqui *Colossoma macropomum* (Cuvier 1818), this study aimed to analyze the morphometric data and expression of myogenic regulatory factors (MRFs) in the white and red muscle from tambaqui obtained from clear and black Amazonian water systems. All of the MRF transcript levels (*myod*, *myf5*, *myogenin*, and *mrf4*) were significantly lower in the red muscle from black water fish in comparison to clear water fish. However, in white muscle, only the *myod* transcript level was significantly decreased in the black water tambaqui. The changes in MRFs gene expression in muscle fibers of tambaqui from black water system provide relevant information about the environmental influence as that of water systems on gene expression of muscle growth related genes in the *C. macropomum*. Our results showed that the physical and chemical water characteristics change the expression of genes that promote muscle growth, and these results may be also widely applicable to future projects that aim to enhance muscle growth in fish that are of substantial interest to the aquaculture.

## 1. Introduction

The Amazon basin is considered the largest drainage system in the world, consisting of thirteen principal rivers, of which it is possible to highlight the Amazon, Negro, Solimões, and Tapajós rivers, and many different environments and different water types may be observed throughout this region [1, 2]. Amazonian water system may be classified, according to its physical and chemical characteristics, as being either white water, which originates in the Andes (Amazon and Solimões Rivers), clear water, which originates from ancient land of massive central of Brazil and Guyana (Tapajós river), or black water, which originates in sandy sediments of Tertiary of Central Amazon (Negro River) [3, 4]. Amazonian white water has a high mineral concentration, a neutral pH (6.5 to 7.0), and a high conductivity. The clear water type has a variable pH (4.5 to 7.0) and a relatively low conductivity. And the black water type has an acid pH (3.0 to 5.0) and contains a high concentration of humic acid, which accounts for its dark color. Black water also has a low concentration of minerals and a marked absence of calcium and magnesium ions [4] and has been characterized as having a dearth of nutrients, low penetration of sunlight, lack of aquatic plants [5, 6], and prevalence of fish species that are small in size, which means a miniaturization process of different fish species found in this water system. Previous studies have shown that there are approximately 40 species of miniature fishes in Amazonian black water [7, 8].

Despite the environmental variations found among the different Amazonian water types, some fish species, such as tambaqui (*Colossoma macropomum*), may be found in all three aquatic environments [9–11]. This species occupies a prominent position in domestic fisheries and aquaculture, in part because of its flavorful filet, which is largely composed of skeletal muscle white fibers [9, 12, 13].

Skeletal muscle displays a great deal of plasticity and may have different morphofunctional characteristics as a result of the influences of various intrinsic and extrinsic factors including temperature, nutrition, fasting, and photoperiod. Previous studies have suggested that these factors may influence the expression of genes that encode proteins that regulate myogenesis and muscle growth, such as myogenic regulatory factors (MRFs) [14–20].

The MRF family comprises the transcripts of the *myod*, *myf5*, *myogenin*, and *mrf4* genes [21]. During embryogenesis, *myod* and *myf5* are known as primary factors that are expressed in myoblasts during the proliferation phase, whereas *myogenin* and *mrf4* are expressed in myoblasts that are in developmental stages in which fusion and differentiation into immature muscle fibers take place [22–24].

Postembryonic muscle growth occurs via the activation, proliferation, and differentiation of undifferentiated myoblasts in muscle fibers, and these events are initiated and controlled by the differential expression of the MRFs [24]. Skeletal muscle hyperplasia and hypertrophy are growth mechanisms regulated by the sequential expression of MRFs. *Myod* and *myf5 *control the determination of myogenic lineage, and they also regulate myoblast activation and proliferation [25, 26]. *Myogenin* and *mrf4* act at the myoblast differentiation stage, during which myotubes fuse to form new myofibers [22, 27]. The proliferation of myoblasts and cell hyperplasia (defined as the formation of new myotubes and their subsequent differentiation into new muscle fibers) may be initiated by an elevation in the levels of *myod* and *myf5* expression in the early stages of muscle growth [28]. The expression of *myogenin* and *mrf4* is more abundant in adulthood, and the expression of these MRFs is related to cellular processes that are associated with myoblasts differentiation and muscle fiber hypertrophy (increases in the number of nuclei that promote the synthesis of additional myofibrils) [28].

Considering that MRFs gene expressions are important for muscle growth and that these growth factors can be affected by extrinsic factors, we hypothesized that the Amazonian water system (clear versus black water) would affect the expression of muscle growth-related genes in juvenile tambaqui, *Colossoma macropomum*, found concurrently in more than one Amazonian water system. The aim of the present work was to analyze the patterns of MRFs (*myod*, *myf5*, *myogenin,* and *mrf4*) gene expression in white and red muscle tissues that were isolated from juvenile *C. macropomum* specimens, acquired from Amazonian black and clear water systems. This study may provide evidence that allows us to better understand the mechanisms of environmental influence in the regulation of the gene expression patterns that control muscle development and growth and may be widely applicable to future projects that aim to enhance muscle growth in fish, and it presents a substantial interest to aquaculture.

## 2. Materials and Methods

### 2.1. Animal Samples

A total of 20 adult tambaqui specimens (*Colossoma macropomum*; (Characiformes, Characidae)) were obtained from private fishery stations in the Brazilian states of Pará and Amazonas that presented intensive systems of breeding. The specific fisheries from which we obtained our animals were Costa do Tapará Fish Farm, which is located in the municipality of Santarém, Pará, Brazil, and Fazenda Santo Antônio Fish Farm, which is located in the municipality of Rio Preto da Eva, Amazonas, Brazil.

The two fishery stations from which we acquired the specimens used a carved tank system and had similar feeding (fed with commercial diets with 40% crude protein until satiation, twice a day) and breeding conditions, but Costa do Tapará and Fazenda Santo Antônio used water from the Amazon (clear water) and Urubu (black water) rivers, respectively. The photoperiod and temperature were the same in both conditions because fish were collected in the same period of the year, presenting similar natural conditions. The pH of water systems was 6.0 to 7.0 for clear water and 4.0 to 5.0 for black water. 

In each fish farm, we used fishes from the same breeding and age (three months). The average lengths of the sample specimens from the clear- and black-water environments were 8.47 ± 1.37 and 7.50 ± 0.64 cm, respectively. A nonparametric *t*-test was developed and these data showed a nonsignificative difference between the samples (*P* = 0.064).

Fish specimens were euthanized using benzocaine (0.1 gL^−1^), individually measured (cm), and muscle samples were collected. White and red muscle samples were collected from tambaqui, immediately placed in storage at a temperature of −80°C, and kept frozen until RNA extraction. In addition, some of the samples of white muscle tissue were subsequently fixed in buffered formalin and used for morphological analysis.

All experiments were approved by the Ethics Committee at the Bioscience Institute, UNESP-São Paulo State University (Protocol no. 178-CEEA).

### 2.2. Morphological and Morphometric Analysis

Fish muscle is predominantly composed of white muscle, which accounts for approximately 60% of the total muscle mass, and the use of it is widely commercialized. For muscle morphology evaluation and fiber diameter analysis, white muscle samples (*n* = 10 for fish from clear water and *n* = 10 for fish from black water) were obtained from deep lateral line region, located close to the cranial region of the right side of the fish body. All samples were taken from the same anatomical position.

The samples were subjected to morphometric analysis to characterize their muscle growth characteristics. White muscle samples were removed, immediately fixed in buffered formalin, and embedded in historesin. Histological sections of 4 *μ*m in thickness were obtained and subsequently stained with haematoxylin and eosin [29] to evaluate the morphometric patterns of the muscle fibers. An image analysis system (Leica Qwin, Wetzlar, Germany) was used to determine the smallest diameter of each muscle fiber within a fiber population, and these diameters were used to evaluate the patterns of hypertrophic and hyperplastic growth of white muscle. In accordance with a classification scheme that was proposed by Veggetti et al. [30], the muscle fibers were categorized as belonging to one of three different size classes depending on their diameters (the three classifications were diameters of <20 *μ*m, diameters of 20–50 *μ*m, and diameters of >50 *μ*m). The mean diameter of the fibers within each diameter class (<20 *μ*m, 20–50 *μ*m, and >50 *μ*m) was calculated, as were the appropriate standard deviations. The frequencies with which fibers of each diameter class were observed were also calculated and were measured as percentages. The high frequency of fiber diameters in <20 *μ*m class can be related to a predominant hyperplastic growth process and the high frequency of fiber diameters in >50 *μ*m class indicates a predominant hypertrophic growth process. 

### 2.3. RNA Isolation and Reverse Transcription

Approximately 100 mg of white and red muscle fragments was mechanically homogenized with 1 mL of the TRizol Reagent (Invitrogen Life Technologies, Carlsbad, CA, USA) and total RNA extraction was conducted in accordance with the manufacturer's protocol. The total RNA samples were incubated with DNase I-Amplification Grade (Invitrogen Life Technologies, Carlsbad, CA, USA) to remove any contaminating DNA. The RNA samples were eluted in RNase-free water, after which a small aliquot of each sample was loaded into an agarose gel to verify the integrity of the RNA. The samples were then quantified (Thermo Scientific NanoDrop 1000 Spectrophotometer) by measuring their optical densities (ODs) at a wavelength of 260 nm. RNA samples were considered sufficiently pure when a 260/280 nm OD ratio of ≥1.80 was obtained.

Two micrograms of total RNA was reverse transcribed using a High Capacity cDNA Archive Kit (Applied Biosystems, Foster City, CA, USA), and the characteristic reaction mixture contained 10 *μ*L of reverse transcriptase buffer (10X RT Buffer), 4 *μ*L of dNTP (25X), 10 *μ*L of Random Primers (10X), 2.5 *μ*L of MultiScribe Reverse Transcriptase (50 U/*μ*L), and 2.5 *μ*L of Recombinant Ribonuclease Inhibitor RNAseOUT (40 U/*μ*L).

### 2.4. RT-PCR, Sequencing, and Sequence Analysis

The cDNA samples were amplified using primer pairs that were specific for the amplification of 18 S rRNA genes (18 S1: 5′-TACCACATCCAAAGAAGGCAG-3′; 18 S2: 5′-TCGATCCCGAGATCCAACTAC-3′) [31] and the four MRFs (MyoD, Myf5, Myogenin, and MRF4) (Table 1). The constitutively expressed 18 S rRNA was used as a positive control in assays of the integrity of the RNA that had been extracted from each tissue sample. Each cDNA amplification reaction mixture consisted of 0.2 *μ*g of cDNA (10%), 0.2 mM of each primer, 25 mM MgCl_2_ PCR buffer, 0.2 mM of dNTPs, and 0.2 unit of platinum *Taq* DNA polymerase (Invitrogen Life Technologies, Carlsbad, CA, USA), all of which were incorporated into a final volume of 25 *μ*L. The RT-PCR procedures were conducted in accordance with the protocol that was described by Kobiyama et al. [32]. After amplification, the amplification products (10 *μ*L) were fractionated on a 1.5% agarose gel, stained with Gel Red (Life Technologies, Carlsbad, CA, USA), and visualized under UV light (Hoefer UV-25). The molecular weights of the amplified fragments were assigned on the basis of comparison with a 1 Kb DNA ladder (Invitrogen Life Technologies, Carlsbad, CA, USA).

The RT-PCR products were purified and were subsequently subjected to automated sequencing using an ABI 377 Automated DNA Sequencer (Applied Biosystems, Foster City, CA, USA) and a DYEnamic ET Terminator Cycle Sequencing kit (GE Healthcare Life Sciences). The procedures were performed in accordance with the manufacturer's instructions and the same primers that were described in the previous section were used (Table 1). Database searches for the relevant nucleic acid sequences were performed using the BLAST/N [33] tool available through the National Center for Biotechnology Information (NCBI) website (http://blast.ncbi.nlm.nih.gov/Blast.cgi). Sequence alignments were obtained via a Clustal-W function [34], and the consensus sequences were determined manually.

### 2.5. Quantitative RT-PCR and Statistical Analysis

Quantitative RT-PCR (qRT-PCR) was performed using an ABI 7300 Real-Time PCR System (Applied Biosystems, Foster City, CA, USA) and a Power SYBER Green PCR Master Mix Kit (Applied Biosystems, Foster City, CA, USA), which was used in accordance with the manufacturer's instructions. Standard reaction mixtures (25 *μ*L) were assembled using 12.5 *μ*L of Power SYBER Green PCR Master Mix 2x, 2 *μ*L of each primer (5 *μ*M), 2 *μ*L of template cDNA that had been treated with DNase I (Invitrogen Life Technologies, Carlsbad, CA, USA) (20 ng/*μ*L), and 6.5 *μ*L of ultrapure water. The primers for the MRFs were specifically designed using the Primer Express software program v.2.0 (Applied Biosystems, Foster City, CA, USA), and they were based on the DNA sequences that had previously been obtained (Table 1). Template cDNA was subjected to a 1 : 10 dilution, and the cDNA samples were replaced with DEPC water in the negative controls. Real time assays were conducted in duplicate. A total of 40 amplification cycles were performed, and each cycle consisted of heating the samples to 94°C for 15 seconds followed by cooling them to 60°C for one minute. The amplification and dissociation curves that were generated using version 4.0 of the 7300 System/Sequence Detection software program (Applied Biosystems, Foster City, CA, USA) were used to analyze the gene expression data. The qRT-PCR signals were normalized to a segment of the 18 S rRNA housekeeping gene using the 18 S3 and 18 S4 primers (5′-CGG AAT GAG CGT ATC CTA AAC C-3′; 5′-GCT GCT GGC ACC AGA CTT G-3′, resp.) that had been designed on the basis of the consensus sequences for this gene that has been shown to be common among several fish species [31, 35, 36]. 

The amplifications of genes that were related to *myod*, *myogenin*, *myf5*, and *mrf4* resulted in sequences that were used as templates for designing new sets of more specific primers. Then, these primers were used in qRT-PCR reactions that aimed to draw comparisons between the patterns of MRF expression in the muscle tissue samples from the *C. macropomum* specimens, acquired from different Amazonian environments (clear and black water). The qRT-PCR primer products were sequenced and the nucleotide sequences were submitted to BLAST/N tool [33], in order to confirm the high percentage of similarity with the interest gene sequences from this database (GenBank accession numbers: *Piaractus mesopotamicus, *FJ686692, FJ810421; *Tetraodon nigroviridis*, AY616520, DQ453127; *Oreochromis niloticus* × *O. mossambicus*, FJ907953; *Danio rerio*, AF318503, AF270789; *Sternopygus macrurus*, AY396565, DQ059552; *Cyprinus carpio*, AB012881, AB012883).

Standard curves for the target and reference genes that were created on the basis of assuming a linear relationship between the Ct value and the log of the starting cDNA quantity showed acceptable slope values of between −3.8 and −3.3 [37]. These standard curves were obtained by using serial dilutions of the cDNA samples.

The Ct values were used to calculate a relative gene expression value for each transcript using the 2^−ΔΔCt^ method. Using this method, data were recorded as the fold-change transcript levels normalized to both the reference gene and the calibration sample [38].

Kruskal-Wallis tests (nonparametric) that were followed by Dunn's multiple comparisons tests (between genes) were used to compare the patterns of gene expression (*myod*, *myogenin*, *myf5*, and *mrf4*) within fish from a specific type of water. Mann-Whitney *U* tests (nonparametric) were used to make comparisons of the expression patterns of each of the genes in fish from the two different aquatic environments [39]. Differences were considered significant when the *P* value was <0.05 and 95% confidence intervals were used.

## 3. Results

### 3.1. Morphometric Analysis

The morphological analysis of white muscle tissue from *Colossoma macropomum *specimens showed a typical pattern of polygonal or round muscle fibers and multinucleated fibers with peripheral nuclei.

For morphometric analysis, it was used 06 animals from clear water and 10 from black water. The number of fibers analyzed was related to the number of fish evaluated: 756 fibers for clear water and 1207 fibers for black water. For fiber type diameter calculation, we used a compound microscope attached to a computerized imaging analysis system (Leica Qwim, Germany) and measured around 100–150 muscle fibers/fish randomly. The muscle fibers were distributed in a mosaic pattern, and the fibers were of several different diameters that were classified into three diameter-based categories (<20, 20–50, and >50 *μ*m in diameter).

**Figure 1:**
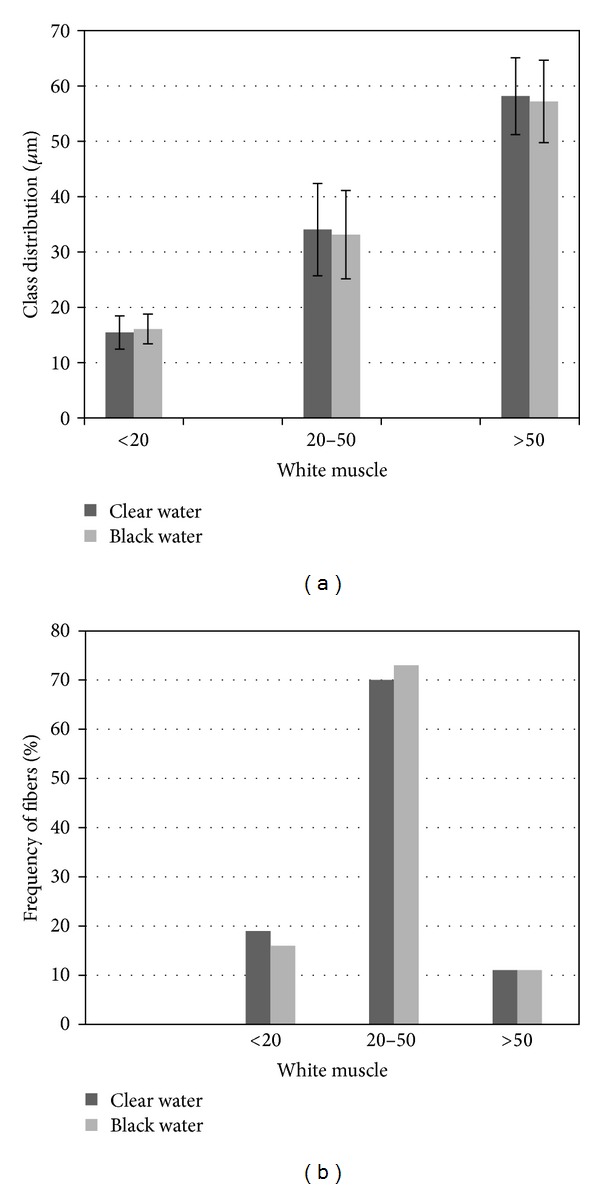
Distribution (a) and frequency (b) of white muscle fibers of *Colossoma macropomum *from clear and black Amazon waters, according to the diameter class classification.

The same pattern of fiber diameters distribution was observed when the white muscle fibers from clear water fish were compared with the muscle fibers from the black water fish. Moreover, the percentages of fibers in each diameter class were similar in the white muscle tissues of both types of fish (Figure 1). It is worth noting that the average lengths of the specimens from the two different aquatic environments were equally similar. The clear water specimens presented a mean length of approximately nine centimeters, and the black water specimens presented a mean length of approximately seven centimeters.

### 3.2. *Myod*, *myf5*, *Myogenin*, and *mrf4* mRNA Expression

 The partial amplifications of the genes that encode *myod*, *myf5*, *myogenin*, and *mrf4* generated evident fragments that were subsequently visualized via electrophoresis in 1.5% agarose gels. The amplified sequences resulted in fragments with lengths of 178, 448, 634, and 176 base pairs (bps) for the four MRF genes, respectively. Comparisons among the sequences that were obtained for white and red muscles did not show any base substitution within the fragments that were analyzed.

DDBJ/EMBL/GenBank database searches and analyses of the degrees of identity of the *Colossoma macropomum *MRF transcript fragments indicated that all of them were similar to related MRF sequences that have been identified for other species of fish, such as *Piaractus mesopotamicus* (FJ686692.1, FJ81042.1), *Tetraodon nigroviridis* (AY616520.1, AY576805.1), *Sternopygus macrurus* (AY396565.1, DQ059552.1), *Cyprinus carpio* (AB012881.1, AB012883.1), and *Danio rerio* (NM_131576.1, BC165074.1). These data represent the first partial descriptions of MRF transcripts that have been derived from the skeletal muscles of *C. macropomum*.

The relative expression levels of the *myod*, *myf5*, *myogenin*, and *mrf4* mRNAs were evaluated on the basis of qRT-PCR result. The data were normalized using the results of a similar analysis of a gene constitutively expressed control (18 S rRNA) in accordance with the 2^ΔΔCt^ method.

**Figure 2:**
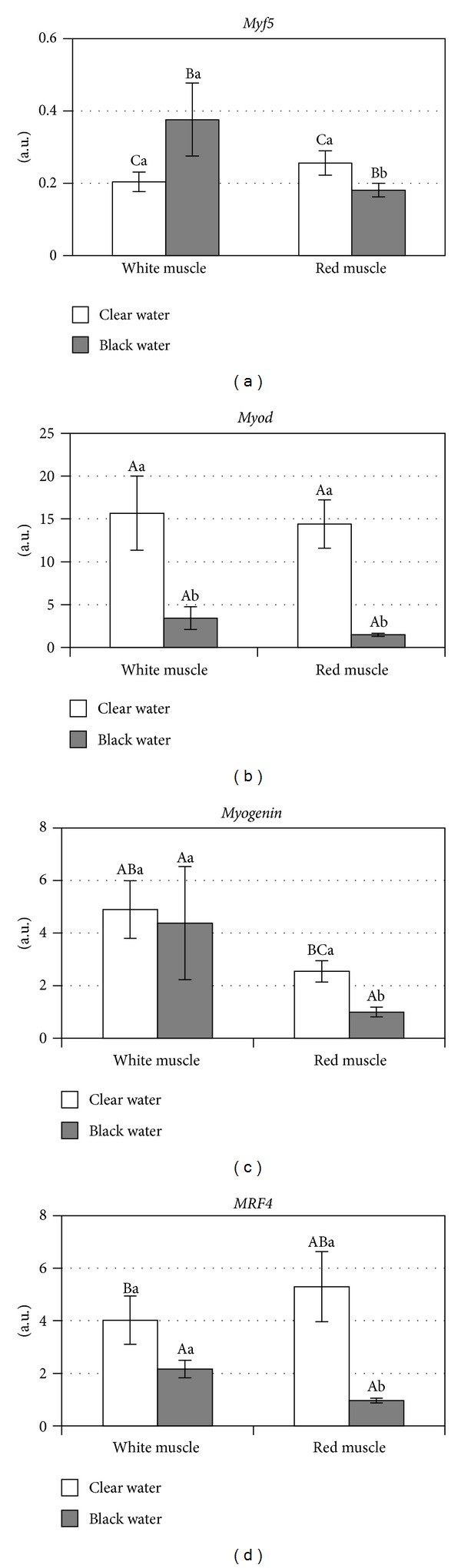
Comparisons of MRFs (*Myf5*, *Myod*, *Myogenin*, and *MRF4*) expression in Amazonian water types (clear and black waters). Upper case: statistical analysis of MRFs (*myf5*, *myod*, *myogenin*, and *mrf4*) transcript levels from white and red muscles. Lower case:   statistical analysis of MRFs (*myf5*, *myod*, *myogenin*, and *mrf4*) transcript levels from clear and black waters.

No significant differences were found when comparing the transcript levels of the various MRFs (*myf5*, *myogenin*, and *mrf4*) in the two types of skeletal muscles in clear water animals (Figures 2(a), 2(c), and 2(d)). However, comparisons between white and red muscle tissue samples from black water fish found evidence of different levels of *myod*, *myogenin*, and *mrf4* transcripts. Significantly lower levels of these transcripts were found in the red muscle relative to the transcript levels in white muscles (Figures 2(b), 2(c), and 2(d)). Comparisons between the two aquatic environments found evidence of significantly lower levels of *myod* transcripts in the white muscles of clear water *C. macropomum*. In contrast, the expression levels of all MRF transcripts were significantly lower in the red muscles of black water fish (Figure 2). Moreover, the *myf5* mRNA levels were significantly lower than the expression levels of other MRF mRNAs in all of the analyzed muscle samples (Figure 2(a)).

## 4. Discussion

The mean lengths of the fish specimens that were used in our study suggest that we used fish that are still at an early stage of development; these fish can reach maximum lengths of up to one meter when fully grown. Thus, the moderate hyperplasia and severe hypertrophy that we observed corroborate the results of previous studies that have indicated a need for prolonged periods of both hyperplasia (an increase in the number of muscle fibers) and hypertrophy (increases in the sizes/diameters of muscle fibers) in fish species, such as *Colossoma macropomum*, that quickly grow to relatively long lengths. Muscle growth depends on both hyperplastic and hypertrophic mechanisms that remain active for a prolonged period of time; these processes are active from the early stages of development to adulthood in fast-growing fish [40, 41]. As tambaqui is a fast-growing fish, the morphometric scenario that we observed may change over the course of the tambaqui life cycle, and, therefore, both body size and growth stage must be taken into consideration in future studies involving tambaqui skeletal muscle. The predominant hypertrophy has been also observed in a close related fish of the tambaqui, the *Piaractus mesopotamicus* (pacu) [35, 36, 42].

The current work represents the first description of the patterns of MRF gene expression in the skeletal muscles of *C. macropomum* that were acquired from different Amazonian aquatic environments. It is also the first study to make inferences about the various ways that these two water types may play roles in regulating the expression of MRFs and therefore muscle growth in this species. 

During muscle growth in fish, *myod* and *myf5* regulate the activation and proliferation of satellite cell, and *myogenin* and *mrf4* are involved in satellite cell differentiation that results in the formation of new muscle fibers and/or enhancement of preexisting fibers [28]. These genes have a portion of highly conserved sequences that encode a region called basic helix-loop-helix domain (bHLH), which allows these factors to connect to specific DNA sequences (E-box), and promote the expression genes that are specific to skeletal muscles [43–45]. 

There are many differences between the white and red muscle tissues in fish. These include differences in anatomic distribution, contractile and metabolic properties, initial development, and growth dynamics [46]. Either white or red muscle fibers are recruited depending on the distinct needs of the organism regarding the use of skeletal muscle. Specifically, the red fibers have slow contractions and oxidative metabolisms and are associated with slow movements that are related to foraging and migrations habits, whereas the white fibers have fast contractions and glycolytic metabolisms and are associated with higher-speed movements that are related to escape and to the capture of food [47–49]. In contrast to these morphophysiological differences between the two types of muscle, the present study did not find any significant differences between the expression patterns of MRF transcripts in the white muscles comparing to the red muscles in clear water fish. We suggest that the discrepancy between our results may be related to the specific growth phase that was evaluated, as well as the difference in physiological properties of white and red muscles, to better adapt to their ecological environment [36, 50]. 

We observed that the expression levels of *myod*, *myf5*, *myogenin*, and *mrf4* were significantly lower in red muscle from black water fish than red muscle from clear water fish but that only *myod* transcript level was significantly decreased in the white muscle tissues of black water fish. In addition, the red muscle tissue from black water fish had significantly lower levels of both *myogenin* and *mrf4* transcripts in comparison to the white muscle tissue from these fish. 

The reduced levels of *myod* transcript in the white and red muscles and *myogenin* and *mrf4* transcripts in the red muscle of black water *C. macropomum*, in comparison to the levels of these transcripts of clear water fish, may offer a clue to the role of the environment in the regulation of the expression of these genes. We can infer that the black water would adversely affect the expression of *myod*, *myogenin*, and *mrf4*, which would result in the less robust proliferation and differentiation of muscle fibers that would ultimately promote a retardation in the fish muscle growth in this water system. The low expression levels of MRFs in these muscle types may represent a diminished rate of cell proliferation that then interferes with normal growth and less intense turnover of contractile muscle proteins. Considering that white muscle comprises around 70% of the bulk mass, our findings could thereby help us to infer the great potential of black water to induce the miniaturization in tambaqui.

Red muscle tissue is important for the metabolism of the muscle as a whole, because it primarily uses an oxidative metabolic pathway, and for sustaining the movements during migration and foraging habits. The changes observed in the red muscle tissue from black water fish may result in subsequent physiological changes that promote the retardation of red muscle fiber growth (i.e., the presence of fewer muscle fibers and muscle fibers that have smaller-than-normal diameters). Although these characteristics were not observed in the morphometric analysis that was conducted in the present study, our analysis does not rule out the possibility that red muscle fiber growth retardation may occur during later stages of the life cycle of *C. macropomum*. 

The specimens that were included in the present study were still in the early stages of growth, and as tambaqui is a fast-growing fish, this pattern may change over the course of the tambaqui life cycle, and, therefore, both body size and growth stage must be taken into consideration in the present study and in future studies of tambaqui skeletal muscle growth. Considering the reduced MRF expression levels to a less intense turnover of contractile muscle proteins and a less pronounced satellite cell differentiation, we suggest that the morphometric data could reveal a muscle fiber growth retardation occurrence at later stages of development of tambaqui from black water system.

Some studies have linked diet and water temperature with the growth of skeletal muscle in fish and have suggested that these factors may influence the expression of genes that regulate myogenesis and muscle growth, such as *myod*, *myogenin*, *myf5*, and *mrf4* [15, 17, 19, 20]. The black water environment is characterized by a poverty of nutrients, a low penetration of sunlight, and a high incidence of fish species that are small in size, and this phenomenon has been associated with the low concentration of nutrients in Amazonian black water [5–8]. This reported nutritional influence could not be related to the findings of the present study, because fishes were fed artificially, using similar feed. Interestingly, a recent study of a Norwegian salmon species showed that pH of the water is an important environmental factor that causes genetic variation among different stocks of these fish [51]. 

The results of the patterns of expression of MRF genes in *C. macropomum* specimens from different Amazonian aquatic environments may provide evidence that will aid researchers in reaching better understanding of the mechanisms of environmental interference in the regulation of gene expression. Moreover, our findings showed that the physical and chemical water characteristics may change the expression of genes that promote muscle growth, and the control of these water characteristics could establish us direct implications toward strategies that benefit muscle growth and ultimately lead to the improved fish production to the aquaculture industry. Ultimately, studies that are related to both the quantification of the patterns of expression of MRF transcripts in white and red muscle tissues and environmental influences on the regulation of MRF gene expression are still scarce. Most of the studies related to the quantitative analysis of MRF mRNAs that have been published to date use a system of analyzing mRNA levels that is less sensitive than qRT-PCR.

## Figures and Tables

**Table 1:** Primer sequences used for RT-PCR and qRT-PCR reactions of *myod*, *myf5*, *myogenin* (*MyoG*), and *mrf4* amplifications.

Primers	Sequences (5′-3′)	Genes	Design
MDLE	CTAACCAGAGGCTGCCHAAG	*myod *	*Ictalurus furcatus *
MDRI	CATGCCATCWGAGCAGTTGG	*myod *	*Ostariophysi *
Myf5FB	GCACGTGCGGGCTCCTG	*myf5 *	*Danio rerio *
Myf5RE	GGACAAACACTGCAAACTGG	*myf5 *	*D. rerio *
MGLG	TGGAGCTTTTYGAGACCAAC	*myog *	*Cyprinus carpio *
MGRF	AGATTGGCTTGCTCCGAAGA	*myog *	*I. furcatus *
MRF4-3	TACCAAGGAAATGACAGCCCTCCA	*mrf4 *	*Sternopygus macrurus *
MRF4-4	TCAACAATTGAGGAGAGGCGACGA	*mrf4 *	*S. macrurus *
*TambMyoD1 *	GCCTGCAGGGACTCGATGTA	*myod *	C. macropomum*
*TambMyoD2 *	GCTGCCCAAGGTGGAGATC	*myod *	C. macropomum*
*Myf5RT2f *	GGCGCAGGCTGAAGAAGGTCAA	*myf5 *	C. macropomum*
*Myf5RT2r *	GCGCCATCCAGTACATTGAGA	*myf5 *	C. macropomum*
*TambMyoG1 *	GCGCCATCCAGTACATTGAGA	*myog *	C. macropomum*
*TambMyoG2 *	GCTCATGTTCCTGCTGGTTGA	*myog *	C. macropomum*
*F4RT2f *	GTGCTGCGTGTTGATCTGCGT	*mrf4 *	C. macropomum*
*F4RT1r *	AGGGGCTCAATGGCCTGCTT	*mrf4 *	C. macropomum*

*Primers used for RT-qPCR amplifications.
